# Factors affecting willingness to receive a kidney transplant among minority patients at an urban safety-net hospital: a cross-sectional survey

**DOI:** 10.1186/s12882-015-0186-2

**Published:** 2015-11-21

**Authors:** Titilayo O. Ilori, Nosayaba Enofe, Anju Oommen, Oluwaseun Odewole, Akinlolu Ojo, Laura Plantinga, Stephen Pastan, Justin B. Echouffo-Tcheugui, William McClellan

**Affiliations:** Department of Medicine, Renal Division, Emory University School of Medicine, Atlanta, Georgia USA; Department of Radiology and Imaging Science, Emory University School of Medicine, Atlanta, Georgia USA; Department of Medicine, Renal Division, University of Michigan, Ann Arbor, Michigan USA; Emory Transplant Center, Emory Healthcare, Atlanta, Georgia USA; Hubert Department of Global Health, Rollins School of Public Health, Emory University, Atlanta, Georgia USA; MedStar Health, Baltimore, Maryland USA; Department of Epidemiology, Rollins School of Public Health, Emory University, Atlanta, Georgia USA; Renal Division, Emory University School of Medicine, 1639 Pierce Drive, Atlanta GA. Clifton Road, Atlanta, Georgia 30322 USA

**Keywords:** African Americans, Willingness, Kidney transplant, CKD, Perceptions, Attitudes, Knowledge

## Abstract

**Background:**

In the US, African Americans (AAs) are four times more likely to develop end stage renal disease (ESRD) but half as likely to receive a kidney transplant as whites. Patient interest in kidney transplantation is a fundamental step in the kidney transplant referral process. Our aim was to determine the factors associated with the willingness to receive a kidney transplant among chronic kidney disease (CKD) patients in a predominantly minority population.

**Methods:**

CKD patients from an outpatient nephrology clinic at a safety-net hospital (*n* = 213) participated in a cross-sectional survey from April to June, 2013 to examine the factors associated with willingness to receive a kidney transplant among a predominantly minority population. The study questionnaire was developed from previously published literature. Multivariable logistic regression analysis was used to determine factors associated with willingness to undergo a kidney transplant.

**Results:**

Respondents were primarily AAs (91.0 %), mostly female (57.6 %) and middle aged (51.6 %). Overall, 53.9 % of participants were willing to undergo a kidney transplant. Willingness to undergo a kidney transplant was associated with a positive perception towards living kidney donation (OR 7.31, 95 % CI: 1.31–40.88), willingness to attend a class about kidney transplant (OR = 7.15, CI: 1.76–29.05), perception that a kidney transplant will improve quality of life compared to dialysis (OR = 5.40, 95 % CI: 1.97–14.81), and obtaining information on kidney transplant from other sources vs. participant’s physician (OR =3.30, 95 % CI: 1.13–9.67), when compared with their reference groups.

**Conclusion:**

It is essential that the quality of life benefits of kidney transplantation be known to individuals with CKD to increase their willingness to undergo kidney transplantation. Availability of multiple sources of information and classes on kidney transplantation may also contribute to willingness to undergo kidney transplantation, especially among AAs.

**Electronic supplementary material:**

The online version of this article (doi:10.1186/s12882-015-0186-2) contains supplementary material, which is available to authorized users.

## Background

Racial Disparities in access to kidney transplantation is a pivotal issue disproportionately affecting the African American (AA) population. In the US, AAs are 4 times more likely to develop end stage renal disease (ESRD) than whites but half as likely to receive a kidney transplant as whites [1, 2]. Even after referral for kidney transplant evaluation, they face numerous barriers to being placed on the waiting list [3–6].

In those without contraindications, renal transplantation which may occur from a living or cadaveric donor, is considered as the treatment of choice for individuals with ESRD because of benefits such as improved long-term survival and quality of life compared to dialysis [7–12]. Although there are well known benefits of renal transplantation, it may not be suitable for every candidate [13]. Referral for kidney transplantation evaluation must occur before evaluation, wait-listing and transplantation, making the entire process a complex, multilevel, multisystem pathway that may discourage the average patient. There are multiple potential barriers to kidney transplantation occurring at the patient, system and physician levels [11, 14–16].

Patient interest in kidney transplantation is a fundamental step in the referral process, which largely influences the decision-making process of ESRD patients and their providers with regards to the choice of renal replacement therapy. Previous studies of ESRD patients showed that individual preferences, perceptions and socio-demographic factors affect treatment choices and attitude towards renal transplantation [6, 17, 18]. However, to our knowledge, only a few studies have examined these barriers to kidney transplantation among chronic kidney disease (CKD) patients [11, 19]. These prior studies have mainly examined individuals on dialysis [2, 3, 6, 9]. Furthermore, factors associated with the willingness to undergo transplant have been less extensively studied in minority populations [20].

Our aim was to determine the factors associated with the willingness to receive a kidney transplant among a predominantly AA population in an inner-city safety-net hospital-based CKD clinic. We hypothesized that factors, such as physician discussion, knowledge and information about transplantation and patient perceptions on kidney transplantation and attitudes/views on kidney donation would affect wanting to undergo kidney transplantation at a future time.

## Methods

### Population and study design

Patients attending an outpatient nephrology clinic, in a large safety-net hospital, catering to a predominantly minority population in Atlanta, Georgia, were approached to participate in a cross-sectional survey between April and June 2013. Fellows and nephrologists from the Emory University School of Medicine staff this clinic. For this study, we only included individuals who self-reported to have been previously diagnosed with CKD. Excluded from the study were patients who were on dialysis or had received a kidney transplant prior to the study and those who were unable to speak English or unable to fill out the questionnaire because of a debilitating illness or other reasons. Participation in the study was completely voluntary and anonymous and verbal consent was obtained. The Emory University Institutional Review Board approved the study.

### Data collection

#### Study questionnaire

A 40-item self-administered questionnaire was administered to 213 CKD patients by the clinic staff during their routine visit to the nephrology clinic (see Additional file 1). Participants were asked to complete the questionnaire in the waiting room while waiting to see their physician and study personnel were available to answer questions if needed. *“*We developed this questionnaire based on previously based on questions from previously published survey items in the literature used in CKD and hemodialysis populations [3, 20, 21] which were modified to suit our population and supplemented with additional questions that we thought may be useful in this particular population based on expert opinion on factors that influence patient decision regarding kidney transplantation [21]. The questionnaire was pilot tested in a separate convenience sample of 39 CKD patients for face and construct validity, in order to ensure that it was appropriate for our population and could measure the outcome specified in our aims [21]. As a result of the pilot testing, we made modifications for content and to ensure that the questions were easily comprehensible at the fifth grade reading level. We also revised the flow and skip patterns to minimize missing data. The final questionnaire contained domains on knowledge and gaining information on kidney transplantation, perceptions (perceptions on kidney transplantation and perceptions on barriers to kidney transplantation and donation), demographic and social characteristics, attitudes/views to kidney donation and social support. We identified and utilized individual items that best operationalized topics or domains of interest. Our objective was for individual items on the questionnaire to capture certain key concepts and not to be combined to provide a composite score or measure.

The final questionnaire contained domains on socio-demographics, knowledge, attitudes, perception and social support (Additional file 1). A total of about 323 questionnaires were distributed and 213 were filled and returned. We did not collect any further information from individuals who did not turn in their questionnaires or declined participation in the study.

### Measurements and constructs

#### Willingness to undergo kidney transplantation

This was assessed by asking “Would you undergo kidney transplant if you are given a chance when the time comes?” with possible responses of “Yes,” “No,” and “Not sure.” We dichotomized responses as “Yes” or “No/Not sure”. Similar methods of measuring individual willingness have been previously published in the literature [3, 20].

#### Knowledge

Prior knowledge of kidney transplant and the source of this knowledge was assessed using “Have you heard about kidney transplant before?” followed by “If yes, from whom?” Responses included “my doctor”, “friend”, “relative”, “social media, literature, news”, “others – specify”. We categorized participant’s responses into “my doctor” and “any other source(s)”. We also asked participants if their doctor had ever discussed kidney transplant as an alternative to dialysis with them. Participants were asked to rate their knowledge about kidney transplant on a 5 point scale ranging from “no knowledge of it” (1 point) to “well informed” (5 points). We categorized these responses as below average (1–2 points), average (3 points) and above average (4–5 points) knowledge. Participants were asked if they had ever been referred for transplant evaluation, and we used this as a proxy for participant knowledge. Prior studies have utilized similar questions in assessing participant’s knowledge in comparable populations [3, 20].

#### *Attitudes*/Views on kidney donation

Participant attitudes toward kidney transplant were assessed by asking if they would they be able to ask for a kidney donation if they needed a kidney transplant, and if they would attend a class on kidney transplantation (yes vs no), we also asked, “If you had had the opportunity, would you have donated your kidneys?” Similar questions have been used in prior literature to assess participant attitudes [21].

#### Perceptions

Perceptions about kidney transplantation and donation were evaluated by the item “Do you think a living person can donate a kidney to patients needing it?” (yes vs no). We also assessed perceived barriers to undergoing a kidney transplant among participants unwilling to undergo a kidney transplant if given a chance when the time comes by asking them to rank, on a four-point Likert-scale, the level of importance attributed to each of the barriers. Perceived barriers assessed are reported in Table 2. Finally, we further evaluated participant’s perception by asking if kidney transplantation would affect their quality of life compared to dialysis [3, 20]. Possible responses were “it will not affect the quality of life”, “improve the quality of life”, “decrease the quality of life”, “I don’t know”. Responses were categorized as correctly answered (improve quality of life) vs. incorrect or did not know.

#### Demographics and social characteristics

Participants age, sex, race, religion, highest education, income, marital status, health insurance, and employment status were assessed to report socio-demographic characteristics. Participants who reported being employed full-time or part-time were recorded as “employed”. Type of health insurance was recorded as “Medicaid”, “Medicare”, “Private Insurance” and “More than one type of insurance.” Household income was also sub-categorized to reflect income class as “$25,000 or less”, “above $25,000 up to $50,000”, “above $50,000” and “other”.

### Statistical analysis

Characteristics of patients were presented by willingness to undergo kidney transplant. Categorical variables were described using numbers and percentages; frequencies of Likert scale and ordinal responses were also reported. Chi-square and Fisher’s exact tests were used to compare categorical variables. Multivariable logistic regression analysis was used to determine factors associated with willingness to undergo a kidney transplant, with model selection performed utilizing backward elimination. Covariates previously documented to be related to the outcome, with plausible behavioral and biologic relationships or with a statistically significant association with the outcome in unadjusted models were included. Variables that remained in the model at the 0.15 significance level were entered into a final logistic regression model. A priori confounders including age, sex, income and insurance status were forced into the final model, and collinearity was assessed using variance inflation factors (VIF). All statistical analyses were performed using SAS 9.3 analytic software and the statistical significance threshold was set at the two tailed level of 0.05.

## Results

### Participant characteristics by willingness to undergo kidney transplantation

#### Socio-demographics

A total of 213 CKD patients participated in this survey and about half were middle-aged. The respondents were primarily AA and mostly female. About half of the participants had completed a high school education/GED, a third reported having some college or graduate education, 44.3 % were unemployed and 70.4 % had an income below $25,000/year. Interestingly, 54.2 % of participants had some form of medical insurance with the highest proportion on Medicare (47.0 %). Overall, 53.9 % of participants were willing to undergo a kidney transplant but there were no statistically significant associations between participant’s sociodemographic characteristics and willingness to undergo a kidney transplant on bivariate analysis (Table 1).

**Table 1:** Baseline characteristics of participants by willingness to undergo kidney transplant

Characteristic	Willingness to undergo Kidney transplant			
	*n*	Yes	No	*P*-value
Age (%)				0.519
18-39	24 (11.3)	13 (11.7)	10 (10.5)	
40-59	110 (51.6)	61 (55.0)	46 (48.4)	
60 and above	79 (37.1)	37 (33.3)	39 (41.1)	
Gender (%)				0.531
Female	117 (57.6)	63 (58.9)	49 (54.4)	
Race (%)				
African American	192 (91.0)	102 (91.9)	86(91.5)	0.565^!^
Other	19 (9.0)	9(8.1)	8(8.5)	
Marital Status (%)				0.523
Married	48 (23.4)	25(22.9)	21(23.6)	
Single	90 (43.9)	51(46.8)	37(41.6)	
Widowed	32 (15.6)	18(16.5)	12(13.5)	
Divorced	35 (17.1)	15(13.8)	19(21.4)	
Income (%)				0.599^!^
25 K^∞^ or less	143 (70.4)	75 (71.4)	65 (70.7)	
Above 25 K up to 50 K^∞^	13 (6.4)	9 (8.6)	4 (4.4)	
Above 50 K	7 (3.5)	3 (2.9)	3 (3.3)	
Other^#^	40 (19.7)	18 (17.1)	20 (21.7)	
Education (%)				0.129
< high school	40 (19.1)	15 (13.8)	23 (24.5)	
High school	99 (47.1)	53 (48.6)	43 (45.7)	
College and above	71(33.8)	41 (37.6)	28 (29.8)	
Employed (%)				0.227
Employed	37 (18.4)	24(22.9)	12(13.3)	
Unemployed	89 (44.3)	45(42.9)	42(46.7)	
Retired	75 (37.3)	36(34.3)	36(40.0)	
Insured (%)				
Yes	111 (54.15)	60 (55.1)	50 (54.4)	0.921
Type of insurance				0.900^!^
Medicaid	32(27.8)	18(30.0)	14(25.9)	
Medicare	54(47.0)	27(45.0)	27(50.0)	
Private insurance	8(7.0)	5(8.3)	3(5.6)	
More than one insurance	21 (18.3)	10(16.7)	10(18.5)	
Preference of living or deceased donor (%)				<.001^!^
Deceased kidney	1(0.6)	1(1.0)	0 (0)	
Living kidney	56(32.0)	39(38.2)	16(22.2)	
I don’t want one	21(12.0)	3(2.9)	1(25.0)	
No preference	97(55.4)	59(57.8)	38(52.8)	
Willingness to undergo Kidney transplant (%)		111(53.9)	95(46.1)	

^!^Fishers-Exact chi-square; *P-value < 0.05; ^∞^25 K = $25,000, 50 K = $50,000; ^#^others = “Don’t know/not sure” or “prefer not to answer”. Note: Percentages may not add up due to rounding. *P*-values less than 0.001 were reported as < .001

#### Perceptions about transplantation

Among participants unwilling to undergo a kidney transplant, response rates for questionnaire item assessing perceived barriers to willingness to undergo kidney transplantation ranged from 27.4 to 33.7 %. Half of the respondents reported that distrust for physicians was important or very important in their unwillingness to undergo a kidney transplant (Table 2). Similarly, a larger proportion of respondents reported that transplant surgery complications (74.2 %) and other surgical concerns (75.6 %) strongly influenced their decision not to undergo a kidney transplant. About 60 % of respondents reported that financial concerns strongly influenced their decision not to undergo a kidney transplant. There was no significant difference in demographic characteristics of participants who did not want to be transplanted and did not respond to any of the questions asked (those with missing data for all perceived barrier questions) and those who responded to at least one of the questions except by income status (*P* = 0.0435. A higher proportion of participants reporting income greater than $25,000 and up to $50,000 were more likely to respond to at least one question assessing perceived barriers to kidney transplant.

**Table 2:** Participant’s perception towards barriers to kidney transplant among participants unwilling to undergo kidney transplantation *n* = 95

	Total (*n*)	Frequency (%)
I don’t trust the doctors *(n = 30)*	95	
*Not important/Somewhat important*		7 (23.33)
*Important/Very important*		15 (50.00)
*Don’t know*		8 (26.67)
*Missing*		65 (68.42)
Religious concerns *(n =* 26*)*	95	
*Not important/Somewhat important*		15 (57.69)
*Important/Very important*		7 (26.92)
*Don’t know*		4 (15.38)
*Missing*		69 (72.63)
Complications from transplant *(n =* 31*)*	95	
*Not important/Somewhat important*		3 (9.68)
*Important/Very important*		23 (74.19)
*Don’t know*		5 (16.13)
*Missing*		64 (67.37)
Surgical concerns-pain, fear *(n =* 29*)*	95	
*Not important/Somewhat important*		2 (6.90)
*Important/Very important*		22 (75.86)
*Don’t know*		5 (17.24)
*Missing*		66 (69.47)
I don’t want somebody else’s organ *(n =* 30*)*	95	
*Not important/Somewhat important*		13 (43.33)
*Important/Very important*		10 (33.33)
*Don’t know*		7 (23.33)
*Missing*		65 (68.42)
I don’t think I’ll ever need it. I feel healthy *(n =* 29*)*	95	
*Not important/Somewhat important*		11 (37.93)
*Important/Very important*		10 (34.48)
*Don’t know*		8 (27.59)
*Missing*		66 (69.47)
Financial concerns *(n =* 32*)*	95	
*Not important/Somewhat important*		6 (18.75)
*Important/Very important*		19 (59.38)
*Don’t know*		7 (21.88)
*Missing*		63 (66.32)

In bivariate comparisons (Table 3), participants willingness to undergo a kidney transplant was associated with the perception that a kidney transplant will improve the quality of life compared to dialysis (*P* < 0.001) and a living person can donate a kidney to a person needing it (*P* <0.001).

**Table 3:** Patient knowledge, attitude and perception by willingness to undergo renal transplant

	Willing to undergo kidney transplant			
	Total	Yes	No	*P*-value
*Knowledge*				
Heard about kidney transplant (Prior knowledge), n (%)				0.004^*^
*Yes*	138 (68.7)	84(77.8)	53(58.9)	
*No/Not sure*	63 (31.3)	24(22.2)	37(41.1)	
Physician discussed transplant with patient, n (%)				0.931
*Yes*	35 (17.9)	19(17.9)	15(17.4)	
*No/Not sure*	161 (82.1)	87(82.1)	71(82.6)	
Ever referred for transplant evaluation, n (%)				0.073^!^
*Yes*	7 (3.6)	5(4.9)	1(1.1)	
*No*	176 (90.7)	94(92.2)	79(89.8)	
*Don’t know/Not sure*	11 (5.7)	3(2.9)	8(9.1)	
Self-reported knowledge on kidney transplant, n (%)				0.008^!*^
*Below average*	158 (79.8)	77(72.6)	77(87.5)	
*Average*	28(14.1)	18(17.0)	10(11.4)	
*Above average*	12(6.1)	11(10.4)	1(1.1)	
*Attitude*				
Willing to attend a class about kidney transplant, n (%)				<.001^*^
*Yes*	112 (55.2)	76(71.0)	33(35.9)	
*No*	27 (13.3)	12(11.2)	15(16.3)	
*Don’t know/Not sure*	64 (31.5)	19(17.8)	44(47.8)	
Willingness to donate kidneys, n (%)				<.001^*^
*Yes*	76 (38.0)	55(50.9)	20(22.5)	
*No*	33 (16.5)	17(15.7)	15(16.9)	
*Not sure*	91 (45.5)	36(33.3)	54(60.7)	
Would you ask for a kidney donation (%)				0.038
*Yes*	102(56.0)	66 (62.9)	36 (47.4)	
*No*	80 (44.0)	39 (37.1)	40 (52.6)	
*Perception*				
Beliefs on whether a living person can donate a kidney (Perceptions on living kidney donation), n (%)				<.001^!^
*Yes*	159(77.6)	102(91.9)	57(61.3)	
*No*	8 (3.9)	2(1.8)	5(5.4)	
*Don’t know/Not sure*	38(18.5)	7(6.3)	31(33.3)	
Perception of quality of life after kidney transplant, n (%)				<.001
*Correct answer (improve quality of life)*	85(45.2)	62(60.8)	22(26.5)	
*Incorrect answer/don’t know*	103(54.8)	40(39.2)	61(73.5)	

^!^Fischer’s-Exact chi-square. *P-value < 0.05. Note: Percentages may not add up due to rounding. *P*-values less than 0.001 were reported as < .001

#### Knowledge and attitude/views towards transplantation and kidney donation

A larger fraction of participants (68.7 %) reported they had previously heard about kidney transplant (Table 3). Only 17.9 % of the total participants reported having ever discussed kidney transplantation as an alternative to dialysis with their doctor. Referral for kidney transplant evaluation occurred in 3.6 % of participants. Finally, the vast majority (79.8 %) of participants rated their knowledge about kidney transplantation as “below average”. Over half (55.2 %) of the total participants were willing to attend a class on kidney transplant and 38 % were willing to donate a kidney if they had had an opportunity (Table 3).

Compared to those not willing to undergo a kidney transplant, participants willing to undergo a kidney transplant were more likely to have heard about kidney transplantation (77.8 % vs 58.9 %, *P* = 0.004), be willing to attend a class about kidney transplant (71.0 % vs 35.9 %, *P* < 0.001), willing to ask for a kidney donation (62.9 % vs 47.4 %, *P* = 0.038) and had a positive attitude toward kidney organ donation (50.9 % vs. 22.5 %, *P* < 0.001) but were less likely to rate their knowledge of kidney transplant as below average (72.6 % vs 87.5 %, *P* = 0.008) in bivariate comparisons.

### Association of participant characteristics with willingness to undergo kidney transplant

Multivariable adjusted analysis revealed four characteristics of participants strongly associated with willingness to undergo a kidney transplant (Table 4). Compared to those who did not know if a living person could donate a kidney, participants who knew that a living person could donate a kidney to a person needing it were more willing to undergo a kidney transplant (OR 7.31, CI: 1.31–40.88). Participants who were willing to attend a class about kidney transplant were also more likely to be willing to undergo a kidney transplant (OR = 7.15, CI: 1.76–29.05). Participants who thought getting a kidney transplant would improve their quality of life compared with dialysis were about 5 times more likely to be willing to undergo a kidney transplant compared with participants who did not think so or didn’t know (OR = 5.40, 95 % CI: 1.97–14.81). Finally, among participants who had heard about kidney transplant, the source of information (other sources vs. physician) was significantly associated with participant’s willingness to undergo kidney transplantation. (OR =3.30, CI: 1.13–9.67).

**Table 4:** Factors associated with positive attitude toward renal transplantation

	Adjusted odds ratio	95 % Confidence interval	
Kidney transplant will affect your quality of life compared with dialysis			
*Correctly answered vs. incorrect or didn’t know*	5.40	1.97	14.81
Willing to attend a class about kidney transplant			
*Yes vs. No*	7.15	1.76	29.05
*Not sure vs. No*	0.97	0.23	4.08
Source of information about kidney transplant			
A*ny other source(s) vs. Participant’s doctor*	3.30	1.13	9.67
Self-reported Kidney transplant knowledge			
*Average and above vs. Below Average*	2.67	0.81	8.84
Beliefs on whether a living person can donate a kidney			
*Yes vs. Don’t know*	7.31	1.31	40.88
*No vs. Don’t know*	15.23	0.79	294.29

Model adjusted for age, sex, income and insurance

Age, sex, household income and insurance status were not statistically significantly associated with participant willingness to undergo a kidney transplant on adjusted multivariable analysis.

## Discussion

Our study investigates one of the preliminary steps of kidney transplantation, assessment of patient’s interest as measured by willingness to undergo transplantation, and also examines factors associated with the willingness to undergo kidney transplantation. We found that the key factors that influence the willingness to receive a kidney transplant in our population after adjusting for confounding variables were the perception that kidney transplantation will improve the quality of life, willingness to attend a class on kidney transplantation, source of information about kidney transplant (others sources vs physician) and the perception that a living person can donate a kidney to someone that needs it. Among our predominantly AA population, sociodemographic factors were not associated with a willingness to undergo kidney transplantation. Although higher self-reported knowledge, willingness to donate one’s own kidneys and prior knowledge about kidney transplant were significantly associated with the willingness to undergo a kidney transplant on crude analysis, they however were no longer significantly associated with the outcome after adjusting for socio-demographic factors and other characteristics.

These findings highlight potentially modifiable factors that are associated with willingness of this CKD population to undergo kidney transplantation. For instance those who correctly answered that kidney transplantation improves quality of life were five times more likely to be willing to undergo a kidney transplant compared with those who answered incorrectly or did not know. This suggests that individual perceptions on the quality of life benefits of kidney transplantation compared with dialysis may play a major role in patient’s decision to obtain a kidney transplant. Therefore, it may be necessary to consider methods to increase patient awareness on the quality of life benefits of kidney transplantation among CKD patients early in the disease process. Compared to those who were not willing to attend a class, individuals willing to attend a class on kidney transplantation were 7.15 times more likely to undergo kidney transplantation. This suggests that a positive attitude toward obtaining education on kidney transplantation may play a somewhat important role in influencing the decision to get a kidney transplant. However, a greater self-reported knowledge of kidney transplantation was not associated with willingness to undergo transplant after adjusting for covariates. Further studies may be needed to explain these observed relationships and also investigate the correlation between individual self-reported knowledge on renal transplantation and actual levels of kidney transplant knowledge in other CKD populations. It was interesting to note that, compared to those who obtained information about kidney transplant only from their physician, those who obtained information from other sources such as friends, family and social media were more willing to undergo kidney transplantation. In addition, we found that only a few CKD patients (17.9 %) had discussed kidney transplantation as an alternative to dialysis with their physicians. Furthermore, physicians' discussion of kidney transplantation with CKD patients was not associated with a willingness to undergo kidney transplantation. These findings may suggest that physicians are not initiating a discussion of kidney transplantation as an option for renal replacement therapy early in the course of the disease process and the quality of patient-physician discussion provided in the outpatient setting may be suboptimal in empowering patient decision to receive a renal transplant. It draws attention to the need for further studies assessing the quality of information obtained on kidney transplantation from physicians. It may imply that physicians may not be equipped with adequate time and know-how to effectively educate patients on renal replacement therapy options. It may also support physician distrust, which may be present in this patient population.

Our findings on improved quality of life as a factor associated with willingness to undergo kidney transplantation are similar to the results seen by Vamos et al. in a Hungarian dialysis population, among whom patients who wanted a transplant expected an improvement in their self-rated health score if they got transplanted [20]. Other studies have also shown that the expectation of improved health on transplant and a decline in health on dialysis is associated with a positive attitude to kidney transplantation [21, 22].

In line with our findings, Finkelstein et al. showed that in a population of CKD patients stage 3–5, there was a limited knowledge of kidney disease and no knowledge of therapeutic choices for ESRD [23]. There have also been studies suggesting the utilization of non-physician medical professionals and alternative educational resources to promote desired health behaviors [24]. Boulware et al. utilized social worker groups and education to improve living donor kidney donation among participants with progressive CKD [24].

While we did not find a significant association between participant’s sociodemographic characteristics and their willingness to undergo a kidney transplant, previous studies have indicated an association between sociodemographic factors and access to transplantation [3, 10, 13, 25, 26]. It is possible that minimal socioeconomic variability in our study population may account for the effects seen in this study. Future longitudinal studies in a more diverse population may be required to further understand this relationship, if any.

Among individuals who were not willing to undergo kidney transplantation, over half of the individuals who responded reported perceived barriers such as fear and pain of surgery and financial concerns as being an important factor influencing their decision and 50 % of individuals reported physician mistrust as an important barrier. This fear of surgery is similar to results seen in ESRD patients [20]. The need for better patient-physician communication in addressing modalities of treatment for ESRD is therefore critical, especially in CKD patients.

There are several potential limitations of this study such as the cross-sectional nature which makes us unable to draw conclusions on the directionality of the associations we found. In addition, the relatively small sample size and the lack of heterogeneity of our population, which was predominantly a low-income AA population not representative of the US CKD population therefore making the results not generalizable to entire CKD population in the US. We also do not have data on subsequent wait-listing and kidney transplantation to assess the association of willingness to undergo transplantation with actual receipt of a transplant. Misclassification due to participant response bias may have occurred. There is a potential for selection bias due to differences between those who were included vs. excluded, and the lack of data on excluded participants does not allow further exploration of this possibility. Finally, as in any observational study, residual confounding is possible. The strengths of this study are that it provides vital information on perceptions and attitudes of a predominantly low income minority population with CKD, and might help inform future interventions to increase willingness to undergo kidney transplantation among this population.

To our knowledge, this study is the first to report willingness to attend a class on kidney transplantation and the source of patient information for kidney transplantation as being associated with willingness to undergo a kidney transplant in pre-dialysis CKD population. Our study examines these factors in a low-socio economic predominantly African American population where only half of the population is uninsured, as opposed to prior studies in well-educated insured populations or in ESRD patients [3, 11].

## Conclusion

In summary, our study demonstrates that individual perception of quality of life benefits of kidney transplantation; willingness to attend class on kidney transplantation; source of information about kidney transplantation; and perceptions on whether a living person can donate a kidney, are significant factors affecting willingness to undergo a kidney transplant particularly among AAs. It is therefore essential that the quality-of-life benefits of kidney transplantation be known to individuals with CKD prior to the onset of dialysis to empower their shared decision-making capacities with respect to choice of ESRD treatment modality. We also propose that stimulating interest in kidney transplantation by promoting multiple information sources such as friends, family, and social media to increase awareness of kidney transplantation and organizing educational classes/outreaches on kidney transplantation presents potentially modifiable factors for which population and clinical interventions can be developed to improve individual willingness to undergo kidney transplantation, particularly among AAs.

## Additional files

Additional file 1:
**Study questionniare.** (PDF 120 kb)

## Abbreviations

AA: African American
CKD: Chronic Kidney Disease
ESRD: End Stage Renal Disease
GED: General Education Development
