# Supplementary material for: Factors affecting willingness to receive a kidney transplant among minority patients at an urban safety-net hospital: a cross-sectional survey
Source: BMC Nephrol. 2015 Nov 21;16:191. doi: 10.1186/s12882-015-0186-2 (PMC4654893; doi:10.1186/s12882-015-0186-2)
Supplement: Additional file 1: — Study questionniare. (PDF 120 kb) [file 12882_2015_186_MOESM1_ESM.pdf]

APPENDIX A: STUDY QUESTIONNIARE

DEMOGRAPHICS

1. What is your age?

   1...........18-29
   2...........30-39
   3...........40-49
   4...........50 -59
   5...........60 -69
   6...........70-79
   7...........80 and above

2. What is your gender?

   1. Male   2. Female

3. What is the highest level of education you have received?

   1. I never went to school
   2. Elementary school 8th grade or less
   3. Grade 9 – 11 some high school
   4. High school 12th grade or GED
   5. college graduate
   6. didn't finish college
   7. post graduate

4. What is your total yearly household or family income from all sources?

   1. $10,000 or under
   2. $10,001-25,000
   3. $25,001- 50,000
   4. $50,001 or more
   5. Don't know/not sure
   6. Prefer not to answer

5. How many people are supported by this income including you?

   1. one (1)
   2. two (2)
   3. three(3)
   4. four(4)
   5. five (5)
   6. Greater than 5

6. What is your race? African American/Black (non-Hispanic)
   1. Hispanic/Latino
   2. Asian
   3. Caucasian/White (non-Hispanic)
   4. Others-please specify ____

7. What is your religion

    1. Christian
    2. Muslim
    3. non denominational
    4. not religious
    5. others- (specify)____

8. What is your employment status?

    1. Employed full time
    2. Employed part time
    3. Unemployed
    4. Retired

9. What is your marital status?

    1. Married
    2. Single
    3. Widowed
    4. Divorced
    5. Not married but living with a partner

The next series of questions will focus on questions about your level of social support.

10. How many friends do you see or hear from at least one a month?

1...........1
2...........2
3...........3
4...........4
5...........5
6...........6 and above

11. How many friends could you call on for help? (by help we mean any form of assistance or support given by a friend such as monetary, emotional, physical or spiritual support)

1...........1
2...........2
3...........3
4...........4
5...........5
6...........6 and above

12. How many family friends could you talk to about personal issues? By personal issues we mean any vital or unsettled problem that affects you. This may be related to finance, health, family, work or any aspect of personal life.

1...........1
2...........2
3...........3
4...........4
5...........5
6...........6 and above

13. How many family members can you call on for help?

1...........1
2...........2
3...........3
4...........4
5...........5
6...........6 and above

14. How many family members can you call on for help? (REPEAT QUESTION)

1...........1
2...........2
3...........3
4...........4
5...........5
6...........6 and above

15. Do you have medical insurance? (The Grady card is not considered health insurance**).**

1. Yes
2. No

16. If Yes, what type? (Indicate all that apply)

1. Medicaid
2. Medicare
3. Private Insurance
4. 1 AND 2.

The next series of questions will focus on knowledge and attitudes towards kidney disease and transplantation.

17. How long have you had kidney disease?

1. One year or less
2. Two years
3. Three years
4. Four years
5. Five years
6. six and above
7. not sure

18. Do you know what percentage of kidney function you have?

1. >60%
2. 30-60%
3. 15-30%
4. <15%
5. I don’t know

19. Have you heard about kidney transplant before?

    1. Yes
    2. No [Skip to question 24]
    3. Don't Know/Not sure [Skip to question 24]

20. If yes, from who?

    1. My doctor
    2. Friend
    3. Relative
    4. Social media, literature, news
    5. Others (please specify)

21. Has your doctor ever discussed kidney transplant as an alternative to dialysis with you?

    1. Yes
    2. No [Skip to question 26]
    3. Don't Know/Not sure [Skip to question 26]

22. How long did your doctor talk to you about transplant?

    1. Didn't talk about transplant.
    2. <15 minutes
    3. 15-30 minutes
    4. Over 30 minutes.
    5. I don't think the time has come yet.

23. Have you ever been referred for transplant evaluation?

    1. Yes
    2. No
    3. Don't Know/Not sure

24. Are you aware of any transplant centers in Atlanta?

    1. Yes
    2. No
    3. Don't Know/Not sure

25. How would you rate your knowledge about kidney transplant?

    1. I have no knowledge of it
    2. Little
    3. Average
    4. Above average
    5. Well informed

26. Do you feel that you need to know more about kidney transplant?

    1. Yes
    2. No
    3. Don't Know/Not sure

27. If there is a class about kidney transplant, would you attend?

1. Yes
2. No
3. I don't know

28. Do you know the level of kidney function when a transplant can be done?

1. When the kidney function is less than 20ml/mi
2. Only after a patient has started dialysis.
3. I don't know

29. Would you undergo kidney transplant if you are given a chance when the time comes?

1. Yes
2. No
3. Don't Know/Not sure

30. If you answered "No" to question 29 above please rank how important these factors are in your decision not to undergo a kidney transplant.

	Not important	Somewhat important	Important	Very important	Don't know
a. I don't trust the doctors **TRUST DOCTORS**	1	2	3	4	8
b. I need more time to think and learn about it **MORE TIME**	1	2	3	4	8
c. Religious concerns **RELIGIOUS CON**	1	2	3	4	8
d. Complications from transplant **COMPLICATIONS**	1	2	3	4	8
e. Surgical concerns-pain, fear **SURG CONCERNS**	1	2	3	4	8
f. I don't want somebody else's organ in my body **SOMEBODY ORGAN**	1	2	3	4	8
g. I don't think I'll ever need it. I feel healthy **FEEL HEALTHY**	1	2	3	4	8
h. Financial concerns- not sure how the cost of transplant and medicines will be covered **FINANCIAL CONCERNS**	1	2	3	4	8

i. Other (please specify) ________**OTHER**____

31. Do you think a living person can donate a kidney to patients needing it?

1. Yes
2. No
3. Don't Know/Not sure

32. Would you prefer a living kidney from a living person or a deceased (a person who has previously died) kidney?

1. Deceased Kidney
2. Living Kidney

3. N/A- I don’t want a kidney transplant.
4. No preference

33. If you need a kidney transplant, do you think you would be able to ask someone to donate a kidney to you?

    1. Yes
    2. No [Skip to question 36]

34. If yes, who would you ask?

    1. Close Family (spouse, children, parents, siblings)
    2. Relatives
    3. Friends
    4. All of them Friends or family
    5. Others please Specify ___________________

35. If no, why is that?

    1........................ I am worried of the effects of losing one kidney in them
    2........................ I don’t want my relatives to know that I have kidney disease
    3........................ I don’t have anybody to ask
    4........................ I feel uncomfortable asking something for myself
    5........................ Not sure how the cost of transplant would be covered
    6........................ Others please Specify ___________________
    7........................ More than or equal of any two above responses

36. If you had the opportunity, would you have donated your kidneys?

    1. Yes [Skip to question 39]
    2. No
    3. I’m not sure

37. If no, why?

    1. Fear of surgery
    2. What if my single kidney fail in future
    3. I’m not healthy overall.
    4. My family won’t let me.
    5. Others please Specify ___________________

38. Do you think that a person’s race can affect their chances of getting a kidney transplant?

    1. Yes
    2. No [Skip to question 41]
    3. I don’t know [Skip to question 41]

39.. If you answered yes to question 39 above, what role do you think race plays in getting a kidney transplant?

    1. No role
    2. I think it will help getting a transplant
    3. I think it might delay or limit me from getting a transplant.
    4. I don’t know/ not sure

40. Quality of life refers to it refers to how the individual's wellbeing may be impacted over time by a disease, a disability, or a disorder. How do you think getting a kidney transplant will affect your Quality of life compared with dialysis?

1. Will not affect the quality of life
2. Improve the quality of life
3. Decrease the quality of life
4. I don't know

Thank you for taking this survey.
